# Is the manchester mobility score a valid and reliable measure of physical function within the intensive care unit

**DOI:** 10.1186/2197-425X-3-S1-A553

**Published:** 2015-10-01

**Authors:** D Mcwilliams, G Atkins, J Hodson, M Boyers, T Lea, C Snelson

**Affiliations:** Queen Elizabeth Hospital, Birmingham, United Kingdom

## Introduction

Early and structured rehabilitation programmes have been shown to decrease both critical care and hospital length of stay (LOS) [1] as well as improve functional ability at the point of critical care discharge [2]. At present there is no general or universally accepted method for measuring mobility within the critical care unit or to track rehabilitation progress [3]. The Manchester Mobility Score was developed in 2005 as one such tool to describe the levels of mobility seen within critical care. Since development, the MMS has been used and adapted in several large critical care units within the UK, but has not previously been investigated in terms of validity and reliability

## Objectives

Our aim was to test the validity and reliability of the Manchester Mobility Score (MMS) as a quick and simple tool for monitoring rehabilitation within critical care.

## Methods

This prospective observational study was performed within a large 75 bed, UK based mixed dependency critical care unit. The study was divided into 2 stages: stage one was the inter-rater reliability testing of the MMS and stage 2 was to assess for correlation with another validated measure of function within critical care and explore any relationship with hospital length of stay post critical care discharge.

## Results

Stage 1 - MMS were collected for 111 patients over a 2 day period. All participating physiotherapists and nursing staff reported that the MMS took less than 1 minute to complete and was easy to use. The inter-rater reliability was excellent with all 3 assessors assigning the same MMS score for every patient (Kappa value = 1).

Stage 2 - A total of 53 patients were included in the second stage analysis. The correlation between the MMS and the Barthel Index on critical care discharge was found to be strong (p < 0.001), with the MMS also showing negative association with hospital length of stay (p < 0.001).

## Conclusions

In conclusion, the MMS is feasible, quick and reliable to complete with strong inter-rater reliability and correlation with the Barthel Index at critical discharge. It may also be useful in predicting post critical car length of stay in critical care survivors.

**Figure 1:**
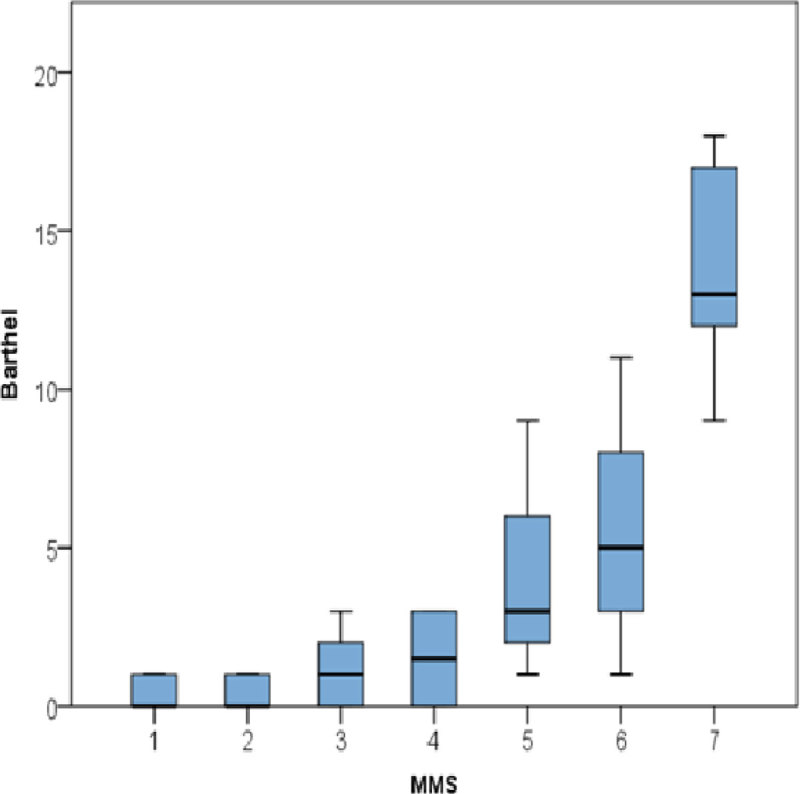
**Correlation between MMS and Barthel scores**.

**Figure 2:**
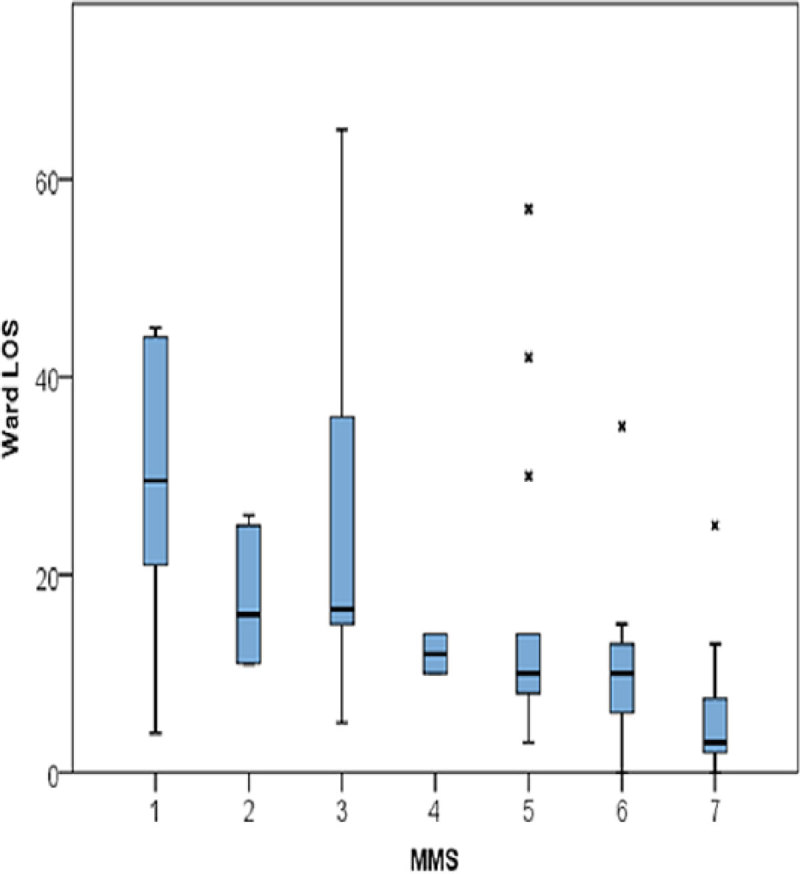
**Correlation between MMS and Length of stay**.
